# Transient Elastography for Detection of Liver Fibrosis in Children With Autosomal Recessive Polycystic Kidney Disease

**DOI:** 10.3389/fped.2018.00422

**Published:** 2019-01-11

**Authors:** Dorota Wicher, Irena Jankowska, Patryk Lipiński, Paulina Szymańska-Rożek, Jakub Kmiotek, Wojciech Jańczyk, Jacek Rubik, Krystyna Chrzanowska, Piotr Socha

**Affiliations:** ^1^Department of Medical Genetics, The Children's Memorial Health Institute, Warsaw, Poland; ^2^Department of Gastroenterology, Hepatology, Feeding Disorders and Pediatrics, Children's Memorial Health Institute, Warsaw, Poland; ^3^Faculty of Mathematics, Informatics and Mechanics, University of Warsaw, Warsaw, Poland; ^4^Department of Nephrology, Kidney Transplantation and Arterial Hypertension, Children's Memorial Health Institute, Warsaw, Poland

**Keywords:** transient elastography, autosomal recessive polycystic kidney disease, congenital hepatic fibrosis, portal hypertension, splenomegaly, thrombocytopenia, esophageal varices

## Abstract

**Introduction:** Congenital hepatic fibrosis (CHF) is invariably present in all patients with autosomal recessive polycystic kidney disease (ARPKD) but is usually clinically asymptomatic. The portal hypertension in the course of CHF develops and progresses over time, so an early detection of liver fibrosis remains crucial.

**Aim:** The aim of the study was to evaluate a predictive value of transient elastography for evaluating liver disease progress in pediatric ARPKD patients.

**Material and Methods:** The study group encompassed 21 pediatric patients with ARPKD and 20 healthy children (control group) from The Children's Memorial Health Institute in Warsaw, Poland. Liver fibrosis was determined by assessing the liver stiffness (LS) with transient elastography (FibroScan®, FS) using size-appropriate probes. In ARPKD group the laboratory findings, results of an abdominal ultrasound examination, and an endoscopic gastroduodenoscopy were also analyzed.

**Results:** Compared with healthy controls, patients with ARPKD had significantly increased median LS values (22 vs. 4.25 kPa, *p* < 0.0001). Based on FS results, ARPKD group was divided into two subgroups: patients (*n* = 5) with LS results suggestive of no fibrosis or minimal fibrosis (LS < 6.9 kPa, METAVIR fibrosis stage 0–1) and patients (*n* = 16) with LS results suggestive of at least significant liver fibrosis (LS ≥ 6.9 kPa, METAVIR fibrosis stage 2–4). In the first subgroup (no fibrosis or minimal fibrosis), all patients had no signs of portal hypertension. In the subgroup with at least significant liver fibrosis, splenomegaly was observed in 87.5% of patients and thrombocytopenia in 69% of patients. An endoscopic gastroduodenoscopy was performed in 15 out of 21 ARPKD patients, nine patients (60%) had esophageal varices. All of these patients had LS results suggestive of at least significant liver fibrosis.

**Conclusions:** TE by FibroScan can be used as an additional method for evaluating liver disease progress in pediatric ARPKD patients.

## Introduction

Autosomal recessive polycystic kidney disease (ARPKD) belongs to the group of congenital hepatorenal fibrocystic syndromes and constitutes a significant cause of renal- and liver-related morbidity and mortality in children (1, 2). Liver involvement in the form of congenital hepatic fibrosis (CHF) is invariably present in all ARPKD patients (3–5).

The liver biopsy is still considered as a gold standard for diagnosis of CHF. Histologically, CHF is characterized by a variable degree of periportal fibrosis and irregularly shaped proliferating bile ducts. The liver disease is usually clinically asymptomatic. The first noted clinical signs include splenomegaly or hepatosplenomegaly which are usually noted accidentally during physical examination. One or more blood cytopenias (as a result of hypersplenism) or slightly elevated serum transaminases could be observed in laboratory analyses. Nonetheless, the portal hypertension develops and progresses over time, so an early detection of liver fibrosis remains crucial (3–5).

Recent studies show that liver stiffness measurements (LSMs) by transient elastography (FibroScan®, FS) have an accurate diagnostic performance for the diagnosis of liver fibrosis in children with selected chronic liver diseases (6–15).

The aim of the study was to evaluate the predictive value of TE by FibroScan for evaluating liver disease progress in pediatric ARPKD patients.

## Materials and Methods

The study group encompassed 21 pediatric patients with autosomal recessive polycystic kidney disease (ARPKD) and 20 healthy children (control group) from The Children's Memorial Health Institute in Warsaw, Poland.

All known ARPKD patients, hospitalized in our Institute, were included in the study, Diagnosis of ARPKD was made based on clinical criteria proposed by Zerres et al. (16). ARPKD group consisted of 13 girls and 8 boys, with age ranged from 5 to 17.5 years (median: 11.9 years). The control group consisted of 20 healthy (without liver and kidney disease) children: 5 girls and 15 boys, hospitalized in our outpatient's clinic, with age similar to study group, ranged from 4.3 to 17 years (median: 9 years).

An informed and written consent was obtained from the study and control groups and also from the parents of the participants in the study. Ethical approval of the study protocol was obtained from the Children's Memorial Health Institute Bioethical Committee, Warsaw, Poland.

Liver stiffness measurements (LSMs) by transient elastography (FibroScan®, FS; Echosens, Paris, France) were performed in all children by the same person (medical doctor). Measurements were made before breakfast or at least 2 h after a big meal. Two different probes were used: S probe (small probe, for thoracic diameter < 45 cm) and M probe (medium probe, for thoracic diameter >45 cm). Quality criteria of TE were the following: IQR (interquartile range) < 25%, number of valid measurements −10, LS reliability assessed as IQR/M ≤ 25%. Results were presented in kPa (FibroScan's scale: 0–75 kPa). The liver stiffness (LS) value cut point >6.9 kPa was used to detect a significant liver fibrosis (METAVIR fibrosis stage 2–4). Fitzpatrick et al. suggested this LS value cut point as an optimal for significant fibrosis in pediatric chronic liver disease (9).

In ARPKD group the laboratory findings (including the erythtrocyte, leukocyte, and platelet counts), results of an abdominal ultrasound examination and an endoscopic gastroduodenoscopy (presence of esophageal varices) were also analyzed. Diagnosis of splenomegaly was made by ultrasound based on proposed criteria (17). The hypersplenism was diagnosed defined as one or more blood cytopenias in the setting of splenomegaly.

For this small group of patients, data were presented as a median and quartile range values. We compared results obtained in subgroups of patients and in ARPKD. Since the samples were relatively small and the results of FS in some subgroups (for example in patients without splenomegly) did not follow the normal distribution (checked with Shapiro–Wilk test for normality), Mann–Whitney *U*-test was used to detect whether the differences between the subgroups are significant. Results were regarded to be statistically significant at *p* < 0.05. The analysis was performed with Statistica for windows software (version 10, Tulsa, USA).

## Results

The diagnosis of ARPKD in four patients was prenatal, in seven patients was made during the first year of life and in the other 13 patients was made later in childhood (1–9 years).

During ARPKD course, nine patients from our cohort developed the end-stage renal failure (ESRF); eight of them underwent the kidney transplantation (KTx), and one of them required a hemodialysis at the last follow-up time.

The most common finding in the liver ultrasound examination was an increased liver echogenicity, which was noted in 16 (76%) out of 21 patients. Other findings include: hepatomegaly in 11 patients (52%), biliary tract dilatation in five patients (24%), presence of small liver cysts in two patients (10%), cholelithiasis in one patient, and presence of collateral circulation in three (1.5%) patients.

Liver stiffness measurements (LSMs) by FS were done in the group of 21 pediatric patients with ARPKD and 20 healthy children. Compared with healthy controls, patients with ARPKD had significantly higher median LS values (22 vs. 4.25 kPa; upper and lower quartiles were 27.5 and 10.3 kPa for ARPKD group and 4.6 and 3.75 kPa for healthy controls; *p* < 0.0001). Results are summarized in Table 1.

**Table 1:** The characteristics of ARPKD group.

**Pt**	**Sex**	**Age of diagnosis**	**RRT**	**Results of liver ultrasound**	**Splenomegaly**	**Results of EG**	**Thrombocytopenia**	**Age at last follow-up (years)**	**Liver stiffness in FS (kPa)**
1	F	Prenatal	KTx	Hepatomegaly	Yes	2 × EVL	Yes	17	25.1
2	M	2 mo	No	Increased LE, dilated BD	Yes	1 × EVL	Yes	9	12.3
3	F	9 y	KTx	Hepatomegaly	Yes	Normal	No	11.9	16.9
4	M	2.5 y	No	Hepatomegaly, increased LE	Yes	2 × EVL	Yes	12.9	13.3
5	F	No data	KTx	Normal	No	Normal	No	12	6.6
6	F	Prenatal	No	Normal	No	No gastroscopy	No	6.5	4.1
7	F	5 y	No	Normal	No	No gastroscopy	No	11.1	3.6
8	F	No data	KTx	Increased LE, collateral circulation; cholelithiasis	Yes	1 × EVL	No	12.8	43.5
9	M	5 y	No	Hepatomegaly, increased LE, dilated BD, small LC	Yes	No gastroscopy	Yes	6.7	75
10	F	3 mo	No	Heterogenic, increased LE, dilated BD, small LC	Yes	Normal	Yes	14.3	27
11	F	8 y	No	Hepatomegaly, increased LE, dilated BD	Yes	3 × EVL	Yes	12.5	16.5
12	M	1 mo	KTx	Hepatomegaly, increased LE, dilated BD, collateral circulation	Yes	Normal	Yes	9.7	27.5
13	M	3 y	No	Hepatomegaly, increased LE	Yes	Esophageal varices (I grade)	Yes	6.7	75
14	M	5 y	No	Increased LE	No	No gastroscopy	No	16.1	6.2
15	M	10 mo	No	Hepatomegaly, increased LE	Yes	2 × EVL	Yes	14.5	25.7
16	F	No data	No	Hepatomegaly, increased LE	Yes	5 × EVL	Yes	6.6	22
17	F	Prenatal	KTx	Hepatomegaly, increased LE	No	Normal	No	9.6	69.1
18	M	1 mo	KTx	Increased LE, dilated BD	No	No gastroscopy	No	5	5.6
19	F	3 mo	Hemodialysis	Increased LE, additional spleen	No	No gastroscopy	No	8.1	10.3
20	F	1 mo	No	Increased LE, collateral circulation	Splenectomy	5 × EVL	Yes (before splenectomy)	17.5	41.6
21	F	No data	KTx	Hepatomegaly, increased LE, dilated BD	Yes	Normal	No	13.9	26.3

*Pt, patient; M, male; F, female; RRT, renal replacement therapy; EG, endoscopic mo, months; y, years; EVL, endoscopic variceal ligation; KTx, kidney transplantation; LE, liver echogenicity; BD, bile ducts; LC, liver cysts*.

ARPKD group was divided into two subgroups: patients (*n* = 5) with LS results suggestive of no fibrosis or minimal fibrosis (LS < 6.9 kPa, METAVIR fibrosis stage 0–1) and patients (*n* = 16) with LS results suggestive of at least significant liver fibrosis (LS ≥ 6.9 kPA, METAVIR fibrosis stage 2–4). In the first subgroup (no fibrosis or minimal fibrosis), all patients had no signs of portal hypertension (the presence of at least one of the following: splenomegaly, thrombocytopenia, esophageal varices). In the subgroup with at least significant liver fibrosis, splenomegaly was observed in 14 out of 16 patients (87.5%) and thrombocytopenia was observed in 11 out of 16 patients (69%). One of these patients underwent splenectomy and he was excluded from portal hypertension-LS analyses.

An endoscopic gastroduodenoscopy was performed in 15 out of 21 ARPKD patients, nine patients (60%) had esophageal varices. Eight patients had undergone at least one procedure of endoscopic variceal ligation (EVL), two patients required five courses of EVL and three patients had a history of gastrointestinal bleeding. All patients with esophageal varices had LS results suggestive of at least significant liver fibrosis.

The LS results between two subgroups of ARPKD patients presented with splenomegaly (*n* = 16, median stiffness value = 26 kPa, upper quartile = 51.2 kPa, lower quartile = 16.6 kPa) and without splenomegaly (*n* = 5, median stiffness value = 5.6 kPa, upper quartile = 6.4 kPa, lower quartile = 3.8 kPa) were compared; a statistically significant difference between these groups was observed (*p* = 0.000129).

The LS results between two subgroups of ARPKD patients presented with thrombocytopenia (*n* = 10, median stiffness value = 25.4 kPa, upper quartile = 51.1 kPa, lower quartile = 16 kPa) and without thrombocytopenia (*n* = 10, median stiffness value = 8.5 kPa, upper quartile = 39 kPa, lower quartile = 4.9 kPa) were compared; the *p*-value of the test assessing if there are statistical differences between the subgroups was 0.06.

**Figure 1:**
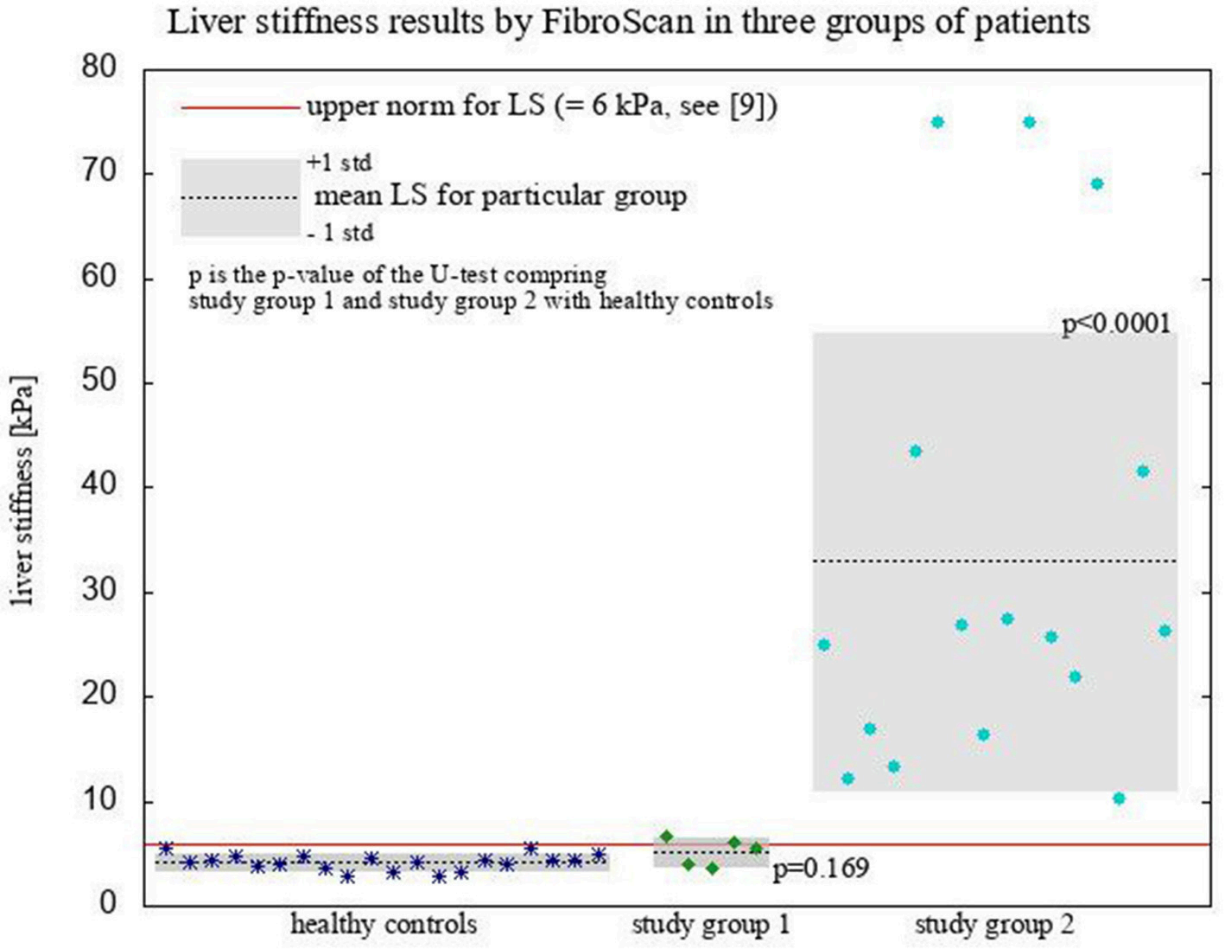
Liver stiffness results by FibroScan.

LS results in presented groups of patients are illustrated on Figure 1.

## Discussion

Congenital hepatic fibrosis (CHF) constitutes an inseparable part of ARPKD. Recent studies suggest that portal hypertension as a result of liver fibrosis could be underdiagnosed in ARPKD patients (3–5).

The liver biopsy is still considered as a gold standard for diagnosis of CHF but it is an invasive procedure and the general anesthesia is required in children (3–5). Thus, non-invasive methods are still developing to facilitate the diagnosis and management of liver disease in ARPKD. The accuracy of transient elastography for detection of liver fibrosis in various chronic liver disease has been shown (6–15). However, up to now, there is only one published study on application of TE in children with polycystic kidney diseases (18). In this study, Kummer et al. compared FS results with a conventional ultrasound in the group of 14 patients with polycystic kidney diseases (seven patients with ARPKD and seven patients with ADPKD). They revealed that in ARPKD patients, the liver fibrosis was detected more often by FS than by conventional ultrasound (100 vs. 57%). They found FS to be a valuable, sensitive, and non-invasive tool for an early detection of liver fibrosis in cystic kidney diseases.

In our study, we analyzed the predictive value of LSMs by FS in the group of 21 pediatric patients with ARPKD. ARPKD patients had significantly higher LS results than healthy volunteers (22 vs. 4.25 kPa, *p* < 0.0001). The signs of portal hypertension (splenomegaly, thrombocytopenia, esophageal varices) were detected only in ARPKD patients with LS results suggesting of at least significant liver fibrosis. The presence of splenomegaly was highly correlated with LS results suggesting of at least significant liver fibrosis (*p* = 0.000129). Routine use of transient elastography by FS could be a reliable tool in non-invasive monitoring of liver disease progress in pediatric ARPKD. Despite lack of correlation between LSMs by FS and presence of esophageal varices, we are convinced that in ARPKD patients presenting with splenomegaly, an endoscopic gastroduodenoscopy should be performed.

Liver stiffness measurements by FS are feasible even in the youngest children (below 2 years of age); however the measurements can be affected by multiple factors such as probe choice, site of measurement, food intake and sedation (19). In addition to fibrosis, the steatosis and hepatitis could also contribute to increased LS results, however CHF patients typically do not develop steatosis or hepatitis (3–5). Therefore, elevated LS results in CHF patients are more specific for fibrosis.

The recent systematic review and meta-analysis showed a good diagnostic performance of ultrasound elastography for evaluating portal hypertension in children (20). It is worth emphasizing that our study was one of the first studies analyzing the role of transient elastography in pediatric ARPKD patients. The strength of the study was that a single observer made the FS measurements and most of affected children underwent endoscopy to compare with FS results. The study had also some limitations. One of the important limitations include a relatively small number of patients and controls included but all known ARPKD pediatric patients, hospitalized in our Institute, were included in the study. The essential limitation is the fact of missing liver biopsy to compare with FS results. However, in patients with typical presentation of liver disease in the course of ARPKD, the liver biopsy is not routinely performed.

Based on our study's results we recommend that in all children that have typical renal ultrasonographic features of ARPKD, the diagnostic work-up should include the examinations assessing liver disease progress, including liver and spleen ultrasonography (with mandatory measurement of the pole distance to assess splenomegaly), laboratory analyses (complete blood count to assess blood cytopenias and liver biochemistry to assess liver function). This diagnostic work-up could also include from now the transient elastography by FibroScan. In selected group of patients, an endoscopic gastroduodenoscopy should be done to assess portal hypertension-related complications.

## Conclusions

Transient elastography by FibroScan can be used as an additional method for evaluating liver disease progress in pediatric ARPKD patients.

## Author Contributions

DW and IJ conception and design of study. DW, IJ, JK, WJ, and JR acquisition of data. DW, IJ, PL, and PS-R analysis and/or interpretation of data. DW, IJ, and PL drafting the manuscript. DW, IJ, PL, PS, KC, and PS-R revising the manuscript critically for important intellectual content. DW, IJ, PL, JK, WJ, JR, PS, KC, and PS-R approval of the version of the manuscript to be published.

### Conflict of Interest Statement

The authors declare that the research was conducted in the absence of any commercial or financial relationships that could be construed as a potential conflict of interest.
